# Baron Hippolyte Larrey

**Published:** 1859-09

**Authors:** 


BARON HIPPOLYTE LARREY.—The son of the distinguished Larrey held, in the recent campaign in Italy, the important post of Director-General of the Army Medical Department in Italy, the same position occupied by his father under Napoleon I. At the battle of Solferino he evinced much boldness and bravery, and had a horse shot under him. Prior to entering upon the campaign, he presented the house in which his father was born to the Parish of Baudéan, and founded an asylum and school for the children of the parish, which he endows with a hundred dollars yearly.

